# Efficacy of Conventional and Novel Tyrosine Kinase Inhibitors for Uncommon *EGFR* Mutations—An In Vitro Study

**DOI:** 10.3390/cells14171386

**Published:** 2025-09-04

**Authors:** Hana Oiki, Kenichi Suda, Akira Hamada, Toshio Fujino, Keiko Obata, Yoshihisa Kobayashi, Kazuko Sakai, Shota Fukuda, Shuta Ohara, Masaoki Ito, Junichi Soh, Kazuto Nishio, Tetsuya Mitsudomi, Yasuhiro Tsutani

**Affiliations:** 1 Division of Thoracic Surgery, Department of Surgery, Kindai University Faculty of Medicine, Osaka-Sayama 589-8511, Japan; hana.oiki@med.kindai.ac.jp (H.O.); a-hamada@med.kindai.ac.jp (A.H.); 154148@med.kindai.ac.jp (S.O.); masito@med.kindai.ac.jp (M.I.); drjsou7@gmail.com (J.S.); mitsudom@med.kindai.ac.jp (T.M.); tsutani@med.kindai.ac.jp (Y.T.); 2 Division of Thoracic Surgery, Izumi City General Hospital, Izumi 594-0073, Japan; 3 Krantz Family Center for Cancer Research, Massachusetts General Hospital Cancer Center, Charlestown, MA 02129, USA; 4 Department of Medicine, Harvard Medical School, Boston, MA 02115, USA; 5 Division of Molecular Pathology, National Cancer Center Research Institute, Tokyo 104-0045, Japan; yoshikob@ncc.go.jp; 6 Department of Genome Biology, Kindai University Faculty of Medicine, Osaka-Sayama 589-8511, Japanknishio@med.kindai.ac.jp (K.N.); 7 Department of Thoracic Surgery, Graduate School of Medicine, Osaka Metropolitan University, Osaka 558-8585, Japan

**Keywords:** personalized treatment, uncommon mutation, molecular targeted therapies, tyrosine kinase inhibitors, acquired resistance, afatinib, osimertinib, lazertinib, Ba/F3 cells

## Abstract

Afatinib and osimertinib are current treatment options for non-small cell lung cancer (NSCLC) patients with uncommon epidermal growth factor receptor (*EGFR*) mutations, although their efficacy is limited. To explore potentially effective drugs for these patients, we evaluated the efficacy of conventional EGFR tyrosine kinase inhibitors (TKIs) and novel third-generation (3G) TKIs using in vitro models. Ba/F3 cells transformed with each of the five most frequent uncommon *EGFR* mutations, Del18 (delE709_T710insD), E709K, G719A, S768I, and L861Q, were used. The growth inhibitory effects of five novel 3G-TKIs, almonertinib, lazertinib, furmonertinib, rezivertinib, and befotertinib, in addition to currently available TKIs, were evaluated. We also explored for secondary resistant mutations to afatinib or osimertinib and TKIs that can overcome these resistances. Afatinib was active against all uncommon *EGFR* mutations tested. The 3G-TKIs were all active against the L861Q mutation and were inactive against the S768I mutation. Furmonertinib and befotertinib showed efficacy against exon 18 mutations (Del18, E709K, and G719A). In the acquired resistance models to afatinib or osimertinib, we found T790M or a novel T725M secondary mutation, respectively, both of which could be overcome by lazertinib. However, some afatinib-resistant cells acquired V769L/M secondary mutations that were refractory to all EGFR-TKIs tested. In conclusion, afatinib exhibited broad activity and some 3G-TKIs showed promising efficacy in the front-line setting. Lazertinib is a potential second-line option after acquisition of resistance to afatinib or osimertinib.

## 1. Introduction

Mutations in the epidermal growth factor receptor (*EGFR*) gene are the most frequent driver mutations in non-small cell lung cancer (NSCLC), especially in patients with East-Asian ethnicity and no history of smoking [1]. Numerous subtypes of *EGFR* mutations have been reported to date [2], and these mutations are usually classified as common mutations (L858R point mutation or exon 19 in-frame deletions) and uncommon mutations (all other mutations). Uncommon mutations are usually detected in about 10% of patients with *EGFR* mutations, irrespective of disease stage [3]. The common versus uncommon classification is useful when considering treatment strategies in advanced-stage settings, because uncommon mutations are usually less sensitive to some of the currently available EGFR tyrosine kinase inhibitors (TKIs) [4]. This observation has been validated in structure-based analysis; many of the uncommon *EGFR* mutations are classified into the P-loop alphaC-helix compressing subtype that is usually insensitive to first- (1G) and third-generation (3G) TKIs, while some (such as L861Q/R) are classified as classical-like *EGFR* mutations [5].

In the Lux-Lung clinical trials [6], afatinib monotherapy demonstrated a progression-free survival (PFS) of 10.7 months (95% confidence interval 5.6–14.7) in patients with NSCLC harboring uncommon *EGFR* mutations (excluding exon 20 insertion- and T790M-positive groups) in the front-line setting. This finding has been validated in the phase III ACHILLES study, which reported superior PFS with afatinib (10.6 months) over platinum plus pemetrexed (5.7 months) in NSCLC patients with uncommon *EGFR* mutations [7]. Osimertinib has also shown some activity against NSCLC in these patients, with phase II studies reporting a PFS of 9.4 (3.7–15.2) months in Japanese patients [8] and 8.2 months (5.9–10.5) in Korean patients [9]. As a result, afatinib or osimertinib are often used in daily clinical practice to treat advanced NSCLC with uncommon *EGFR* mutations. However, the PFS reported for these agents is shorter than that reported for patients harboring common *EGFR* mutations; for example, a PFS of 18.9 months has been reported in patients with common mutations receiving osimertinib [10]. Although the recent CHRYSALIS-2 study (cohort C) demonstrated promising efficacy for amivantamab plus lazertinib in patients with NSCLC harboring uncommon *EGFR* mutations [11] research into single-agent TKI regimens is still warranted because of the high toxicity associated with the amivantamab plus lazertinib combination.

Several novel 3G-TKIs are currently under clinical development, some of which may have activity against uncommon *EGFR* mutations. In addition, some of these new drugs may overcome the acquired resistance that can occur during treatment with currently available TKIs, including afatinib and osimertinib. Here, we used Ba/F3 cell models of NSCLC driven by uncommon *EGFR* mutations to evaluate the efficacy of conventional TKIs and novel 3G-TKIs in the first-line setting and the second line after afatinib or osimertinib treatment failure.

## 2. Materials and Methods

### 2.1. Data Collection from the cBioPortal Database

Data on *EGFR* mutation subtypes and their frequencies in NSCLC were extracted from the cBioPortal database (https://www.cbioportal.org) as of April 2024. We counted the total number of each mutation in exons 18–21 of *EGFR*, excluding L858R point mutation, exon 19 in-frame deletions, and exon 20 in-frame insertions.

### 2.2. Cell Lines and Reagents

The Ba/F3 cell line was purchased from Riken Bio Resource Center (Tsukuba, Japan). Ba/F3 cell lines driven by uncommon *EGFR* mutations, E709K, G719A, exon 18 deletion (delE709_T710insD), and S768I, as well as wild-type human *EGFR* were established in our previous studies [12,13]. In this study, Ba/F3 cells driven by *EGFR* L861Q mutation were established as reported previously [13], and the cells were cultured as previously described elsewhere [12,13]. Human recombinant EGF was purchased from Thermo Fisher Scientific (Waltham, MA, USA). First-generation (1G) EGFR-TKIs (gefitinib and erlotinib), second-generation (2G) EGFR-TKI (afatinib), and 3G EGFR-TKIs (osimertinib, furmonertinib, lazertinib, almonertinib, rezivertinib, and befotertinib) were purchased from Selleck Chemicals (Houston, TX, USA).

### 2.3. Establishment of Ba/F3 Cells Harboring EGFR L861Q Mutation

Ba/F3 cells driven by the *EGFR* L861Q mutation were established as described previously [13]. Briefly, the pBABE retroviral vector with a full-length cDNA fragment of human *EGFR* with the L861Q point mutation was purchased from Addgene (Cambridge, MA, USA). The pBABE construct was co-transfected into gpIRES-293 cells with the pVSV-G vector (Clontech, Mountain View, CA, USA) using FuGENE6 transfection reagent (Promega, Madison, MI, USA). Viral envelopes were generated to produce viral particles. After 48 h of transfection, the culture medium was collected and centrifuged at 1500× *g* for 45 min at 4 °C to concentrate the virus particles. Viral pellets were resuspended in DMEM (Sigma-Aldrich) and stored at −80 °C.

### 2.4. Growth Inhibition Assay

Ba/F3 cells were seeded in 96-well plates at a density of 2500 cells/well. After 24 h incubation, cells were exposed to each TKI at the concentrations determined based on the ranges of clinically achievable drug concentration. After 72 h, 10 µL of the tetrazolium salt WST-8 was added to each well (Cell Counting Kit-8, Dojindo Laboratories, Kumamoto, Japan) and the plates were incubated for an additional 1.5–3 h. The absorbance was read at 450 nm using a multiplate reader (Tecan, Mannedorf, Switzerland), and the growth inhibitory effect was calculated by comparing with DMSO-treated control cells. Growth inhibitory curves were generated and, at the same time, the half-maximal inhibitory concentration (IC_50_) values were automatically calculated using GraphPad Prism 9 (GraphPad Software, Boston, MA, USA). After calculating the IC_50_ values, the sensitivity index (SI), defined as the IC_50_ value divided by the trough concentration of each drug at the recommended dose (IC_50_/Ctrough × 100), was calculated. SIs are more appropriate parameters than IC_50_ values when comparing the efficacy of drugs because clinically achievable blood concentrations vary greatly. SIs have been shown to correlate with the clinical efficacy, such as response rates, of various molecular targeted drugs with one potential cut-off value of 5 [14]. We also evaluated the selectivity index, which was defined as the SI divided by the SI of Ba/F3 cells with wild-type *EGFR*. A smaller value of selectivity index indicates a stronger inhibitory effect on mutant EGFR expressed on tumor cells compared to wild-type EGFR expressed on non-cancerous cells.

### 2.5. Establishment of Resistant Clones to Afatinib and Osimertinib

The *N*-ethyl-*N*-nitrosourea (ENU, Sigma-Aldrich, St. Louis, MO, USA) mutagenesis technique was used to accelerate the establishment of afatinib- and osimertinib-resistant cells, as previously described [15]. Ba/F3 parental cells were initially treated with 100 mg/mL ENU for 24 h. After washing twice with cell culture medium, cells were cultured for 24 h and then plated in 96-well plates (10,000 cells/well) with 10 nM afatinib or 100 nM osimertinib. These drug concentrations were selected to be between the IC_50_ values of uncommon mutations and that of wild-type *EGFR*. We cultured the cells for 14–28 days, with a change in medium every 3 to 5 days. After establishing resistant cells, DNA was extracted using a DNeasy Blood & Tissue Kit (250) (QIAGEN, Venlo, The Netherlands), and secondary *EGFR* mutations were detected by direct sequencing as previously described [16]. We examined all wells with regrowth or randomly selected 12 wells if there were 13 or more wells with confluent cells. If no cells grew in the plate, the drug concentration was reduced by orders of magnitude (5 nM and 2.5 nM for afatinib, and 50 nM and 25 nM for osimertinib), and the same experiments were repeated.

## 3. Results

### 3.1. Frequency of Uncommon EGFR Mutations in cBioPortal Database

**Figure 1:**
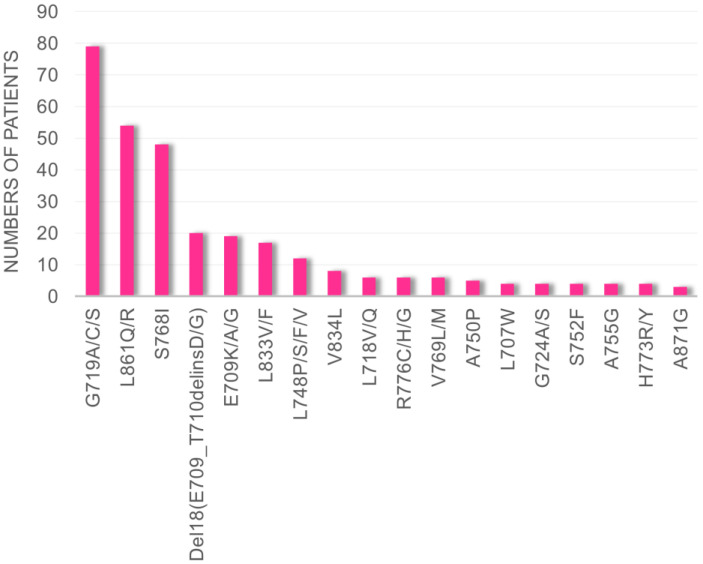
Frequencies of uncommon *EGFR* mutations, excluding exon 20 insertion mutations, reported to the cBioPortal database in patients with NSCLC.

By evaluating data obtained from cBioPortal, we found that G719X, L861X, S768I, Del18 (delE709_T710insD), and E709X were the five most frequent uncommon *EGFR* mutations in patients with NSCLC (Figure 1). Therefore, we decided to evaluate the efficacies of the conventional and novel EGFR-TKIs using Ba/F3 cells harboring these five mutations.

### 3.2. Efficacy of Novel TKIs Against Uncommon EGFR Mutations

**Figure 2:**
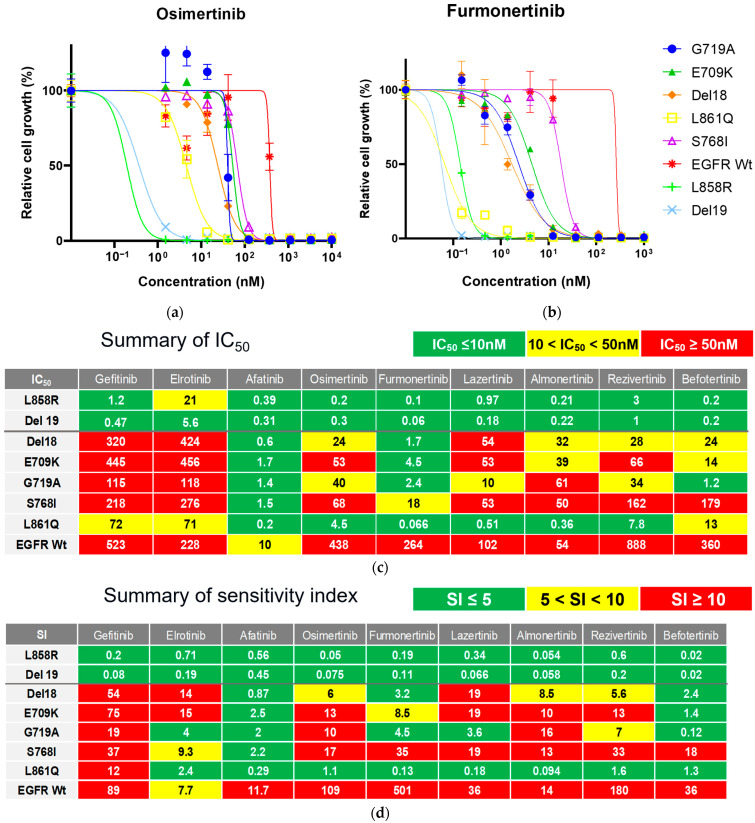
Efficacy of 1G-, 2G-, and 3G EGFR-TKIs against Ba/F3 cells harboring one of uncommon *EGFR* mutations (Del18, E709K, G719A, S768I, or L861Q). (**a**,**b**) Growth inhibitory curves of osimertinib (**a**) and furmonertinib (**b**) against Ba/F3 cells transformed by various common (Del19 or L858R) or uncommon (G719A, E709K, Del18, L861Q, and S768I) *EGFR* mutations. Curves for the other TKIs are shown in Supplementary Figure S1. Data are presented as the mean values from three individual experiments. Error bars indicate the standard deviation. (**c**,**d**) Summaries of the growth inhibitory effects of EGFR-TKIs tested in this study. IC_50_ values (nM) are summarized for each TKI in (**c**). The measured IC_50_ values are color-coded as follows: green (≤10 nM); yellow (10–100 nM); and red (≥100 nM). The SI of each TKI, which was defined as the IC_50_ value/Ctrough × 100, is summarized in (**d**). The calculated SIs are color-coded as follows: green (≤5); yellow (5–10); and red (≥10). The estimated Ctrough value for each TKI and the supporting reference(s) are as follows; gefitinib, 591 nM [17]; erlotinib, 2969 nM [18]; afatinib, 69 nM [19]; osimertinib, 400 nM [20,21,22]; furmonertinib, 53 nM [23]; lazertinib, 281 nM [24,25]; almonertinib, 380 nM [26]; rezivertinib, 493 nM [27,28]; and befotertinib, 1000 nM [29,30]. EGFR, epidermal growth factor receptor; IC_50_, half maximal (50%) inhibitory concentration; SI, sensitivity index; TKI, tyrosine kinase inhibitor.

We evaluated the inhibitory effects of gefitinib and erlotinib (1G-), afatinib (2G-), and osimertinib, almonertinib, lazertinib, furmonertinib, rezivertinib, and befotertinib (3G-TKIs) against Ba/F3 cells harboring one of uncommon *EGFR* mutations to identify TKIs with activity against these mutations. In addition, the efficacies of these drugs were evaluated in Ba/F3 cells harboring wild-type *EGFR* supplemented with 20 ng/mL of human recombinant EGF to assess the side effects of the drugs on non-cancerous cells. Growth inhibitory curves are shown in Figure 2a,b, and in Supplementary Figure S1. IC_50_ values and SIs, which indicate efficacy of drug adjusted with clinically achievable drug concentration, are summarized in Figure 2c and Figure 2d, respectively.

As shown in Figure 2c,d, afatinib showed the greatest inhibitory effect against all tested Ba/F3 cell models with uncommon *EGFR* mutations. Erlotinib was active against L861Q and G719A mutations, and osimertinib was active against the L861Q mutation only when we defined being active by an SI of <5. However, we observed that the ratios of SIs between uncommon *EGFR* mutations and wild-type, hereafter defined as the selectivity index, were smallest in osimertinib (≤15), followed by afatinib (≤17), gefitinib (≤85), and erlotinib (≤200), suggesting that osimertinib may also work clinically, while preserving the phosphorylation of wild-type *EGFR* in non-cancerous cells (Supplementary Figure S2). Among novel 3G-TKIs, we found that all were active against Ba/F3 cells with the L861Q mutation, and all had low efficacy (SIs over 10) against Ba/F3 cells with the S768I mutation. Comparing among the 3G-TKIs, befotertinib was active against all uncommon *EGFR* mutations other than S768I, whereas furmonertinib had the smallest selectivity index (6.9 or smaller) versus osimertinib and afatinib (Supplementary Figure S2).

These results suggest that befotertinib or furmonertinib (especially for higher dosing) are potentially useful 3G-TKIs against NSCLC in patients harboring uncommon *EGFR* mutations. Afatinib and osimertinib are also reasonable treatment options. The potential utility of other drugs (gefitinib, erlotinib, lazertinib, almonertinib, and rezivertinib) could be considered for each mutation variant in accordance with the results summarized in Figure 2d.

### 3.3. Secondary Resistance Mutations to Osimertinib and Strategies to Overcome Resistance

To explore useful TKIs in the second-line setting, we examined potential secondary mutations that may confer acquired resistance to osimertinib using Ba/F3 cells with the three most frequent uncommon mutations (G719A, S768I, and L861Q). After exposing the Ba/F3 cells to ENU, we treated them with osimertinib (starting at 100 nM) for a few weeks.

**Figure 3:**
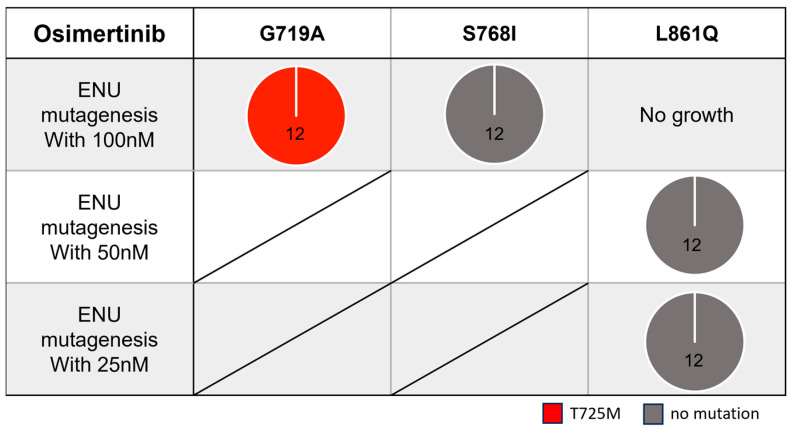
Secondary *EGFR* mutations found in osimertinib-resistant clones established through *N*-ethyl-*N*-nitrosourea (ENU) mutagenesis. Numbers in pie charts indicate established or analyzed clones at each drug concentration (maximum 12 clones).

**Figure 4:**
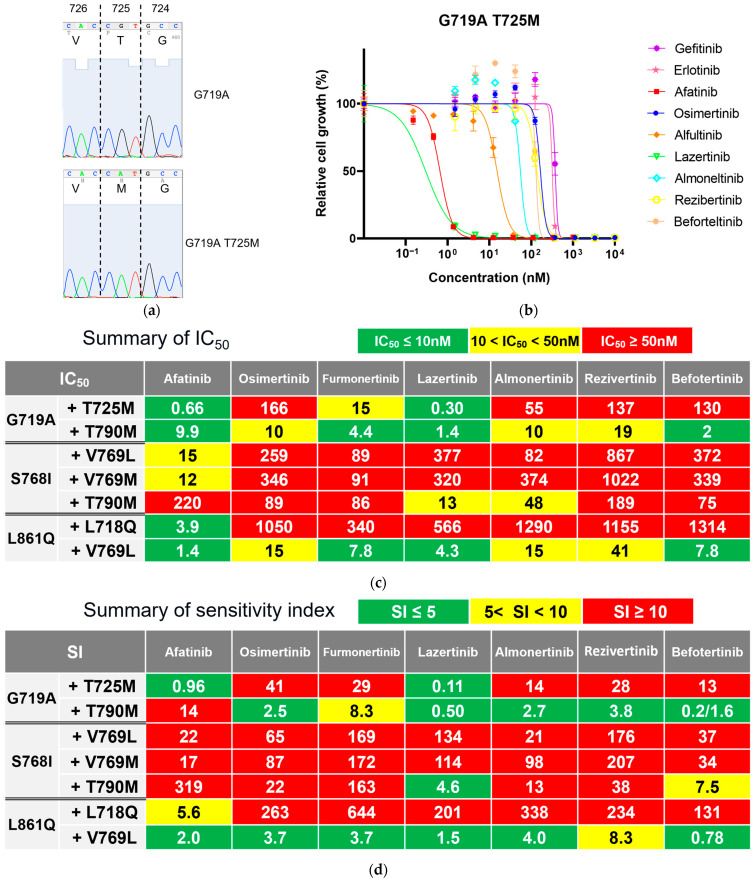
Exploration of TKIs that can overcome T725M and other secondary mutations found in this study. (**a**) Identification of secondary mutation T725M. Sequencing results for parental cells (G719A only) and resistant cells (G719 plus T725M) are shown. (**b**) Growth inhibition curves of each TKI against Ba/F3 cells with G719A plus T725M. Data are presented as the mean values of three individual experiments. Error bars indicate the standard deviation. (**c**,**d**) Inhibitory activities of each TKI used to treat cells with various uncommon *EGFR* mutations were compared using the IC_50_ values (nM, **c**) and SI (IC_50_/Ctrough of each drug × 100, **d**). The measured SI values are color-coded as follows: green (≤5); yellow (5–10); and red (≥10). The estimated Ctrough value for each TKI and the supporting reference(s) are as follows: afatinib, 69 nM [19]; osimertinib, 400 nM [20,21,22]; furmonertinib, 53 nM [23]; lazertinib, 281 nM [24,25]; almonertinib, 380 nM [26]; rezivertinib, 493 nM [27,28]; and befotertinib, 1000 nM [29,30]. EGFR, epidermal growth factor receptor; IC_50_, half maximal (50%) inhibitory concentration; SI, sensitivity index; TKI, tyrosine kinase inhibitor.

We identified the T725M secondary *EGFR* mutation (Figure 3 and Figure 4a) in Ba/F3 cells with the G719A mutation in all viable wells tested. In contrast, we detected no secondary mutations in the kinase domain of the *EGFR* gene in Ba/F3 cells with S768I or L861Q mutations after treatment with osimertinib (Figure 3).

In the growth inhibitory analysis (Figure 4b), Ba/F3 cells with G719A/T725M mutations showed an IC_50_ value of 165.6 nM for osimertinib that was 4.1-fold higher than parental Ba/F3 cells with only the G719A mutation. We observed that Ba/F3 cells with G719A/T725M were insensitive to all novel 3G-TKIs except for lazertinib. In addition, we found that afatinib was active against Ba/F3 cells with G719A/T725M mutations (Figure 4c,d). The selectivity index was also summarized in Supplementary Figure S3.

### 3.4. Secondary Resistance Mutations to Afatinib and Strategies to Overcome Resistance

To explore useful TKIs in the second-line setting after afatinib treatment, we examined potential secondary mutations that may confer acquired resistance to afatinib. After ENU exposure, we treated these Ba/F3 cells with afatinib (starting at 10 nM) for a few weeks.

**Figure 5:**
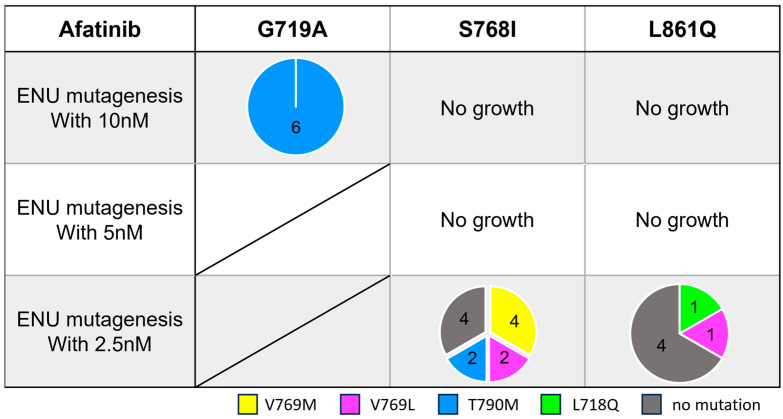
Secondary *EGFR* mutations found in afatinib-resistant clones established through *N*-ethyl-*N*-nitrosourea (ENU) mutagenesis. Numbers in pie charts indicate established or analyzed clones at each drug concentration (maximum 12 clones).

In contrast to osimertinib-resistant cells, we detected several different secondary mutations depending on the activating *EGFR* mutation subtype (Figure 5). In the G719A model, secondary T790M mutation emerged in all six established wells. Ba/F3 cells with G719A/T790M had a 7.1-fold higher IC_50_ value compared with G719A parental cells (Figure 4c). T790M secondary mutation also emerged in cells with S768I mutation at the lowest drug concentration (2.5 nM), and Ba/F3 cells with S768I/T790M had a 146-fold higher IC_50_ value compared with the parental cells. Furthermore, several different substitutions involving V769 were found in Ba/F3 cells with S768I or L861Q mutations.

To explore EGFR-TKIs that may overcome these secondary mutations induced by afatinib, we also evaluated the efficacy of osimertinib and other novel 3G-TKIs. We observed that most of the 3G-TKIs tested were active against Ba/F3 cells with G719A/T790M and S861Q/V769L; however, only lazertinib was effective against Ba/F3 cells with S768I/T790M mutations (Figure 4d). In contrast, Ba/F3 cells with S768I/V769L or S768I/V769M mutations were refractory to all EGFR-TKIs tested. The selectivity index was also summarized in Supplementary Figure S3.

## 4. Discussion

Various studies have reported that afatinib and osimertinib are effective against NSCLCs harboring uncommon *EGFR* mutations; therefore, these drugs are usually the treatment of choice in clinical practice [6,8,9,31,32,33]. However, treatment outcomes with afatinib or osimertinib in NSCLC patients with uncommon *EGFR* mutations are not satisfactory compared with osimertinib treatment of NSCLC in patients with common *EGFR* mutations. Therefore, as a first step in this study, we screened novel 3G-TKIs to identify the most effective agent using in vitro models with uncommon *EGFR* mutations. Because Ba/F3 cells transformed by mutated *EGFR* are solely depend their proliferation and survival on signaling from the mutated (and phosphorylated) EGFR and we have observed correlation between growth inhibitory effects and decreased phosphorylation of driver mutation in some of our previous studies [12], we did not perform Western blotting in this study. As the first result, we found that afatinib was active (SI < 5) against all uncommon *EGFR* mutations, but that osimertinib was less active against many of the uncommon mutations tested. This result is in agreement with a recent pooled analysis comparing the efficacy of afatinib versus osimertinib using propensity score-matching in patients with NSCLC harboring uncommon *EGFR* mutations [34]. However, in our analysis of novel 3G-TKIs, we observed that befotertinib and furmonertinib could be promising TKIs for patients with many of the uncommon *EGFR* mutations, such as L861Q, G719A, or E709K, when considering IC_50_ values, clinically achievable drug concentrations, and the inhibitory effect of wild-type *EGFR* (selectivity index as defined in the Methods section). Although no clear cut-off value was defined for the selectivity index in this study, it would be possible to increase the dosage of TKIs with small selectivity index, such as furmonertinib (Supplementary Figure S2) to enhance the efficacy similar to a previous study of high-dose TKI treatment [35]. Based on these results, we suggest that NSCLC harboring uncommon *EGFR* mutations should not be treated as a single disease; rather, we should determine an appropriate TKI (and appropriate drug concentrations as reported recently [36]) for each mutation subtype.

As the next step, we explored TKIs that can overcome acquired resistance to front-line osimertinib or afatinib. To evaluate potential secondary mutations associated with acquired resistance to osimertinib or afatinib, we used ENU mutagenesis to establish cells with acquired resistance. Although this experimental model has been widely used to detect secondary resistant mutations that may occur in patients who experience acquisition of resistance to TKI(s) [14], as a limitation of this model, we could not rule out the possibility that ENU-induced mutations in genes other than *EGFR* affected the growth inhibition results. However, because Ba/F3 cells with secondary *EGFR* mutations, which were established in this study, showed relatively low IC_50_ values to afatinib and/or lazertinib, we consider that these cells were still dependent on signaling from mutated EGFR. As one of secondary mutations, we detected T725M in Ba/F3 cells with G719A that acquired resistance to osimertinib. Although a machine-learning approach in a previous study has suggested the transforming ability of the *EGFR* T725M mutation [37], this mutation is very rare in clinical practice. By exploring the COSMIC database, we observed that five cases of *EGFR* T725M mutation have been reported (Supplementary Table S1). None had a concurrent G719X mutation, although some patients had concurrent L858R mutation. In addition, through a literature search, we found two studies that reported detecting T725M mutations in tumors after osimertinib treatment [38,39]. Therefore, it is reasonable that the T725M secondary mutation emerged after osimertinib exposure in our in vitro model. Our results also suggest that afatinib or lazertinib could overcome the T725M secondary mutation in *EGFR* G719A-positive patients.

Furthermore, we observed that T790M or V769M/L secondary mutations emerged after afatinib exposure in Ba/F3 cell models with G719A, S768I, or L861Q mutations. We had expected the emergence of the T790M secondary mutation because it has been reported as an acquired resistance mechanism to afatinib in NSCLC among patients with common *EGFR* mutations. Furthermore, it is reasonable that the T790M secondary mutation could be overcome by 3G-TKIs, which were designed to overcome the T790M mutation. Our results showed that lazertinib was the most effective 3G-TKI in this setting.

Among secondary resistant mutations to afatinib, we also observed V769M and V769L mutations in models with S768I and L861Q *EGFR*-activating mutations. Previous studies have reported that the *EGFR* V769M mutation is a frequent *EGFR* germ-line mutation [40,41]; however, it has also been reported that V769M/L mutations have emerged as somatic mutations, usually together with another *EGFR*-activating mutation, as summarized in Supplementary Table S2 [42,43]. Interestingly, V769L often co-exists with the *EGFR* S768I mutation, although the molecular mechanisms responsible for this co-existence are unclear. Two of the affected patients had efficacy data for TKI treatment; neither showed a response to erlotinib or afatinib (Supplementary Table S2). In addition to COSMIC data, additional case studies have reported TKI efficacy against NSCLCs with S768I plus V769L compound mutation; only one patient responded to full-dose afatinib [44], whereas the other two patients showed inherent resistance to gefitinib [45] or lower-dose afatinib [46]. Currently, few case studies have reported the emergence of V769X as a secondary mutation [47]. Therefore, future studies are needed to evaluate the frequency of this mutation after acquiring afatinib resistance in patients with NSCLC harboring uncommon *EGFR* mutations. It should be noted that one patient with the G719A plus V769M compound mutation responded to upfront osimertinib [48].

## 5. Conclusions

Our in vitro study demonstrated that afatinib showed universal activity against various uncommon *EGFR* mutations, while 3G-TKIs, especially furmonertinib and befotertinib, also showed high efficacy against all these mutations, except S768I. Therefore, we suggest that NSCLC with uncommon *EGFR* mutations should not be treated as a single disease but should be treated based on mutation subtype. Furthermore, we detected several on-target resistance mutations of *EGFR*, such as T725M, T790M, and V769M/L, after exposure to osimertinib or afatinib, and our results suggest that lazertinib may overcome some of these secondary mutations.

## Data Availability

The findings of this study are available from the corresponding author (K.S.) upon reasonable request.

## Supplementary Materials

The following supporting information can be downloaded at: https://www.mdpi.com/article/10.3390/cells14171386/s1, Figure S1: Growth inhibitory curves of EGFR-TKIs tested in this study; Figure S2: Summaries of the growth inhibitory effects of EGFR-TKIs tested against Ba/F3 cells harboring an uncommon *EGFR* mutation based on the selectivity index; Figure S3: Summaries of the growth inhibitory effects of EGFR-TKIs tested against Ba/F3 cells with secondary resistant mutation based on the selectivity index; Table S1: T725M mutation reported in lung cancers in COSMIC database (accessed at 31 January 2025); Table S2: V769L/M mutations reported in lung cancers in COSMIC database (accessed at 31 January 2025).

## Abbreviations

The following abbreviations are used in this manuscript:

EGFR	Epidermal growth factor receptor
ENU	*N*-ethyl-N-nitrosourea
IC_50_	Half maximal inhibitory concentration
NSCLC	Non-small cell lung cancer
PFS	Progression-free survival
SI	Sensitivity index
TKI	Tyrosine kinase inhibitor
1G	First-generation
2G	Second-generation
3G	Third-generation
