# Supplementary material for: Efficacy of Conventional and Novel Tyrosine Kinase Inhibitors for Uncommon EGFR Mutations—An In Vitro Study
Source: Cells. 2025 Sep 4;14(17):1386. doi: 10.3390/cells14171386 (PMC12427748; doi:10.3390/cells14171386)
Supplement: Supplementary file 1 [file cells-14-01386-s001.zip › Supplementary Figures.pdf]

Supplementary Figure S1

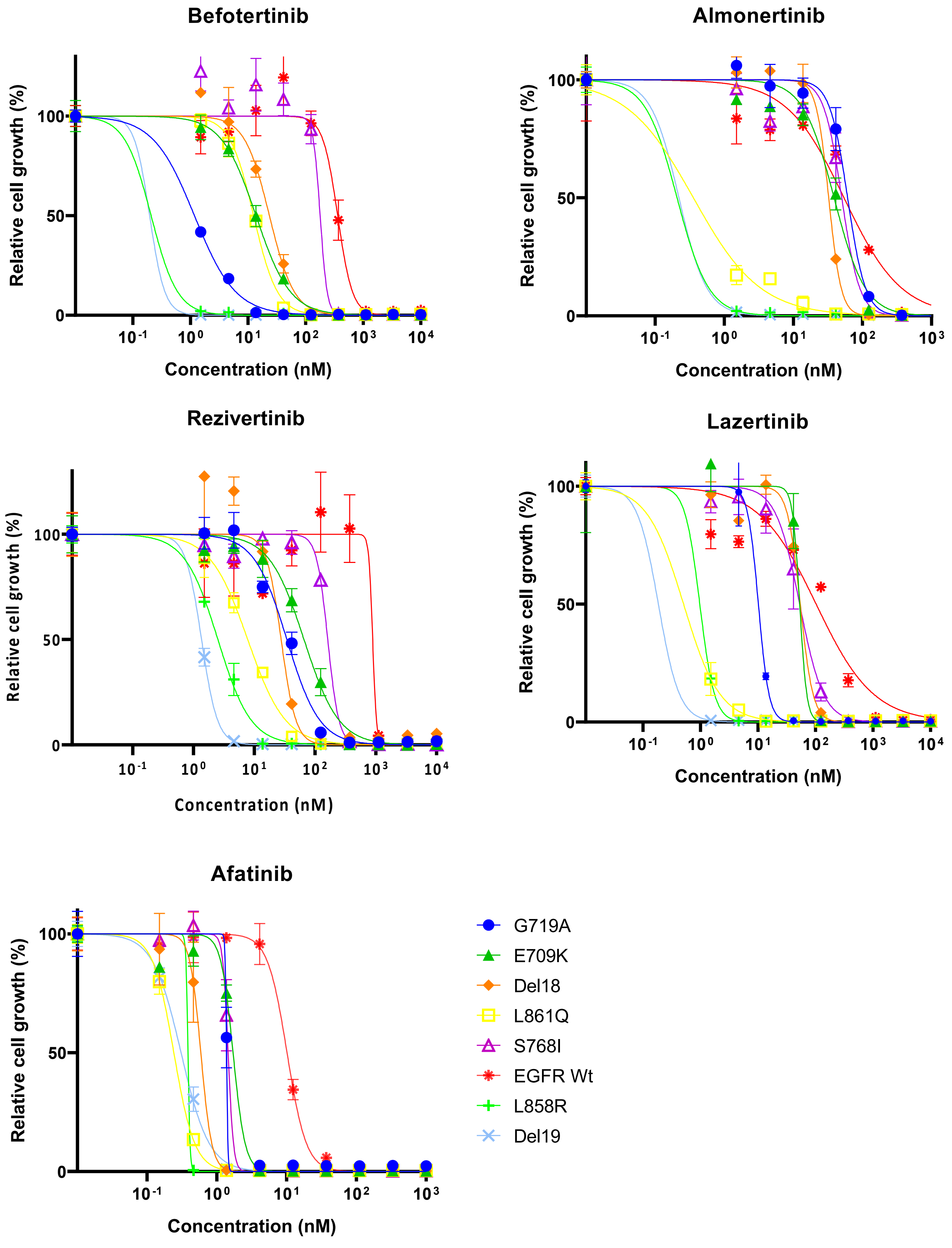

Supplementary Figure S1.
Growth inhibitory curves of EGFR-TKIs tested in this study against Ba/F3 cells transformed by various common (Del19 or L858R) or uncommon (G719A, E709K, Del18, L861Q, and S768I) EGFR mutations. Curves for osimertinib and furmonertinib are included in Figure 2.

Supplementary Figure S2

Selectivity Index	Gefitinib	Elrotinib	Afatinib	Osimertinib	Furmonertinib	Lazertinib	Almonertinib	Rezivertinib	Befotertinib
L858R	0.23	9.3	3.8	0.046	0.038	0.95	0.38	0.34	0.056
Del 19	0.09	2.5	3	0.069	0.023	0.18	0.41	0.11	0.056
Del18	61	186	5.9	5.5	0.64	53	60	3.1	6.5
E709K	85	200	17	12	1.7	52	72	7.4	3.8
G719A	22	52	14	9.2	0.9	9.9	113	3.9	0.33
S768I	42	121	15	15	6.9	52	93	18	50
L861Q	14	31	2	1	0.025	0.49	0.66	0.88	3.5
EGFR Wt	100	100	100	100	100	100	100	100	100

Selectivity Index ≤ 10	10 < Selectivity Index < 50	Selectivity Index ≥ 50

Supplementary Figure S2.
Efficacy of first-, second-, and third-generation EGFR-TKIs against Ba/F3 cells harboring an uncommon EGFR mutation (Del18, E709K, G719A, S768I, or L861Q). Summaries of the growth inhibitory effects of EGFR-TKIs tested based on the selectivity index, which was defined as the sensitivity index (SI) divided by the SI of Ba/F3 cells with wild-type EGFR. The calculated selectivity indices are color-coded as follows: green (≤10); yellow (10–50); and red (≥50).

Supplementary Figure S3

Selectivity Index		Afatinib	Osimertinib	Furmonertinib	Lazertinib	Almonertinib	Rezivertinib	Befotertinib
G719A	+ T725M	6.5	38	5.8	0.29	102	15	36
	+ T790M	97	2.3	1.7	1.4	19	2.1	0.56
S768I	+ V769L	150	59	34	368	151	98	103
	+ V769M	115	79	34	312	692	115	94
	+ T790M	2168	20	32	13	88	21	21
L861Q	+ L718Q	38	241	129	553	2378	130	365
	+ V769L	14	3.3	0.74	4.2	28	4.6	2.2

Selectivity Index ≤ 10	10 < Selectivity Index < 50	Selectivity Index ≥ 50

Supplementary Figure S3.
Exploration of TKIs that can overcome T725M and other secondary mutations found in this study. Summaries of the growth inhibitory effects of EGFR-TKIs tested based on the selectivity index, which was defined as the sensitivity index (SI) divided by the SI of Ba/F3 cells with wild-type EGFR. The calculated selectivity indices are color-coded as follows: green (≤10); yellow (10–50); and red (≥50).
